# QTL Mapping and Diurnal Transcriptome Analysis Identify Candidate Genes Regulating *Brassica napus* Flowering Time

**DOI:** 10.3390/ijms22147559

**Published:** 2021-07-15

**Authors:** Jurong Song, Bao Li, Yanke Cui, Chenjian Zhuo, Yuanguo Gu, Kaining Hu, Jing Wen, Bin Yi, Jinxiong Shen, Chaozhi Ma, Tingdong Fu, Jinxing Tu

**Affiliations:** 1 National Key Laboratory of Crop Genetic Improvement, Hongshan Laboratory, College of Plant Science and Technology, Huazhong Agricultural University, Wuhan 430070, China; sjr2015@webmail.hzau.edu.cn (J.S.); lb15623278513@163.com (B.L.); cuiyanke2021@163.com (Y.C.); zhuocj@webmail.hzau.edu.cn (C.Z.); hukaining@gmail.com (K.H.); wenjing@mail.hzau.edu.cn (J.W.); yibin@mail.hzau.edu.cn (B.Y.); jxshen@mail.hzau.edu.cn (J.S.); yuanbeauty@mail.hzau.edu.cn (C.M.); futing@mail.hzau.edu.cn (T.F.); 2 Institute of Economic Crops, Xinjiang Academy of Agricultural Sciences, Urumqi 830091, China; gyg771746@sohu.com

**Keywords:** rapeseed, flowering time, QTL mapping, RNA-seq

## Abstract

Timely flowering is important for seed formation and maximization of rapeseed (*Brassica napus*) yield. Here, we performed flowering-time quantitative trait loci (QTL) mapping using a double haploid (DH) population grown in three environments to study the genetic architecture. Brassica 60 K Illumina Infinium™ single nucleotide polymorphism (SNP) array and simple sequence repeat (SSR) markers were used for genotyping of the DH population, and a high-density genetic linkage map was constructed. QTL analysis of flowering time from the three environments revealed five consensus QTLs, including two major QTLs. A major QTL located on chromosome A03 was detected specifically in the semi-winter rapeseed growing region, and the one on chromosome C08 was detected in all environments. Ribonucleic acid sequencing (RNA-seq) was performed on the parents’ leaves at seven time-points in a day to determine differentially expressed genes (DEGs). The biological processes and pathways with significant enrichment of DEGs were obtained. The DEGs in the QTL intervals were analyzed, and four flowering time-related candidate genes were found. These results lay a foundation for the genetic regulation of rapeseed flowering time and create a rapeseed gene expression library for seven time-points in a day.

## 1. Introduction

Rapeseed (*Brassica napus*), also known as oilseed rape or canola, is a major oil crop globally and the third-largest source of vegetable oil after soybean and palm (USDA, 2019, https://www.ers.usda.gov/data-products/oil-crops-yearbook/, last accessed date 12 September 2020). It can be used to produce edible oil and industrial materials, and its protein-rich oil meal is used for animal feed. *Brassica napus* (AACC, *n* = 38) was generated from diploid *Brassica rapa* (AA, 2*n* = 20) and *Brassica oleracea* (CC, 2*n* = 18) through natural interspecific hybridization less than 7500 years ago; therefore, it is a relatively young allotetraploid [1]. Rapeseed is classified as winter, spring, and semi-winter types based on vernalization requirement differences. The semi-winter and spring types are the main types planted in China, of which the semi-winter type is primarily planted in the Yangtze River basin and the spring type in the northern region.

Flowering indicates the plant has completed its conversion from the vegetative stage to the reproductive stage, and it is essential for plant reproduction and crop yield formation [2]. By adjusting rapeseed flowering time, it can be adapted to farming systems in different regions to maximize planting area and yield [3,4]. Therefore, flowering time is an important trait to be considered in rapeseed breeding. Rapeseed and *Arabidopsis thaliana* (*A. thaliana)* belong to the Brassicaceae family, are closely related and possibly descended from a common ancestor [5]. The homologous genes from *A. thaliana* and rapeseed have high sequence similarity, and include flowering-related genes [6]. As a model plant, the flowering-time regulation network of *A. thaliana* has been extensively studied, including photoperiod, vernalization, autonomous, aging, gibberellin (GA), ambient temperature, and sugar [7]. 

Quantitative trait loci (QTL) linkage mapping is a classic method widely used in the study of complex quantitative traits in rapeseed, such as silique length and seed weight [8], branch angle [9], and seed glucosinolate content [10]. Some studies on rapeseed flowering time have used QTL linkage mapping. For example, Raman et al. (2012) used the DH population to detect over 20 QTLs controlling flowering time, of which seven were related to the vernalization response and explained 59.4% of the phenotypic variations [11]. Shen et al. (2018) used the phenotypic data of four traits (flowering time, stem diameter, plant height, and branch initiation height) of the DH population in six environments to conduct QTL research, and found that two flowering time QTLs on chromosomes A02 and A07 were colocalized with other three traits [12]. Xu et al. (2021) used F_2_ populations and F_2:3_ families to identify ten flowering time-related QTLs in four environments, of which the one located on chromosome A09 may be a novel QTL [13].

RNA-seq is a powerful tool for transcriptome research and is widely used for gene function studies [14], analysis of biological pathways involved in traits [15], and aiding QTL candidate gene identification [16,17]. Jones et al. (2018) used RNA-seq of leaves and shoot apices at different stages of rapeseed development to reveal various spatiotemporal expressions in flowering-time gene homologs [18]. Jian et al. (2019) analyzed the flowering-time QTLs of a RIL (recombinant inbred line) population and used RNA-seq to obtain the DEGs in the QTL intervals, determining that eight differentially expressed flowering-time genes may be candidate genes [16]. RNA-seq at multiple time-points in a day could reveal the expression pattern of genes during a day and it has been widely used in research on rhythmically expressed genes in animals [19] and plants [20], including the related species (*Brassica rapa)* of rapeseed [21,22]. However, rhythmic transcriptome analysis has not yet been reported in rapeseed. 

Flowering time is the main factor determining the regional adaptability of rapeseed, and it has a significant impact on yield. Many studies have investigated rapeseed flowering-time QTLs, but there are few studies on QTLs that have regional specificity and that exist stably in all environments. The use of RNA-seq at multiple time-points in a day to analyze rapeseed flowering time has not yet been reported. In this study, we constructed a high-density genetic linkage map, analyzed consensus QTLs in three environments, and found one specific QTL in the semi-winter growing region and a QTL existing stably in all environments. Combined with RNA-seq analysis at multiple sampling time-points, we identified potential candidate flowering-time genes that could enrich the understanding of the rapeseed flowering-time regulatory network and provide a genetic basis for advances in rapeseed flowering-time research. We constructed an expression library for genes in rapeseed, presenting the expression dynamics of genes at seven points in a day and laying the foundation for the study of the functional differentiation of paralogous genes and gene expression regulatory networks in rapeseed.

## 2. Results

### 2.1. Analysis of Phenotypic Data in Double Haploid (DH) Population and Parents

Bing409 (B409) is a photoperiod-sensitive restorer line. In the same environmental conditions, as the day length becomes shorter, the flowering time is prolonged compared with that of other materials (Table S1). The parents’ and DH populations’ flowering time data were obtained at three locations in China from 2016 to 2018. As illustrated in Table 1, the flowering time data from the parents showed significant differences in the three environments. Zhongshuang8hao (ZS8) bloomed 13, and 24.4 days earlier than B409 in the semi-winter type growing region (17 SG, and 18 KM). However, in the spring type growing region (16 HZ), B409 bloomed 2.56 days earlier than ZS8 (Table 1). The day length in the spring type region was longer than in the semi-winter type region during the rapeseed growth period (Figure S1A). The vernalization time of semi-winter type region was longer than that of the spring type region (Figure S1B). Consequently, the flowering time of the two growing region showed obvious variations between the parents and DH population. The DH population flowering time in the semi-winter type region ranged from 60–159 days and the flowering time in the spring-type region was shortened to 64–87.3 days. 

**Figure 1:**
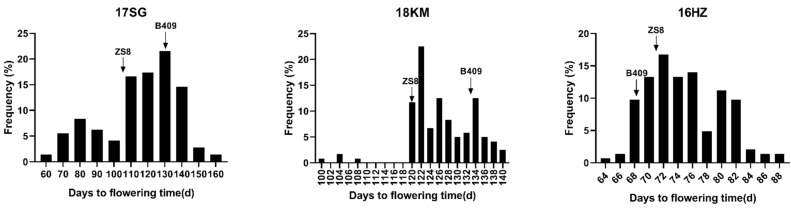
Double haploid (DH) population frequency distribution of flowering-time data in three environments. 17 SG: Shaoguan in 2016–2017; 18 KM: Kunming in 2017–2018; and 16 HZ: Hezheng in 2016. B409: female parent Bing409; ZS8: male parent Zhongshuang8hao. Black arrows represent average flowering time of parents in each environment.

The correlation among flowering time of the three environments demonstrated a significant positive correlation (r^2^ = 0.436–0.807, *p* < 0.0001). Among them, the correlation between the semi-winter regional environments was as high as 0.807 (Table S2). The analysis of variance revealed significant differences in the genotype, environment, and their interaction, indicating that these factors have an important impact on flowering time (Table S3). From the flowering-time frequency distributions in the DH population, we found that the flowering time showed transgressive segregation and continuous distributions (Figure 1). Therefore, the flowering time trait was a typical quantitative trait in the DH population, controlled by a polygene and easily affected by environmental conditions.

### 2.2. DH Population Genetic Linkage Map Construction

**Figure 2:**
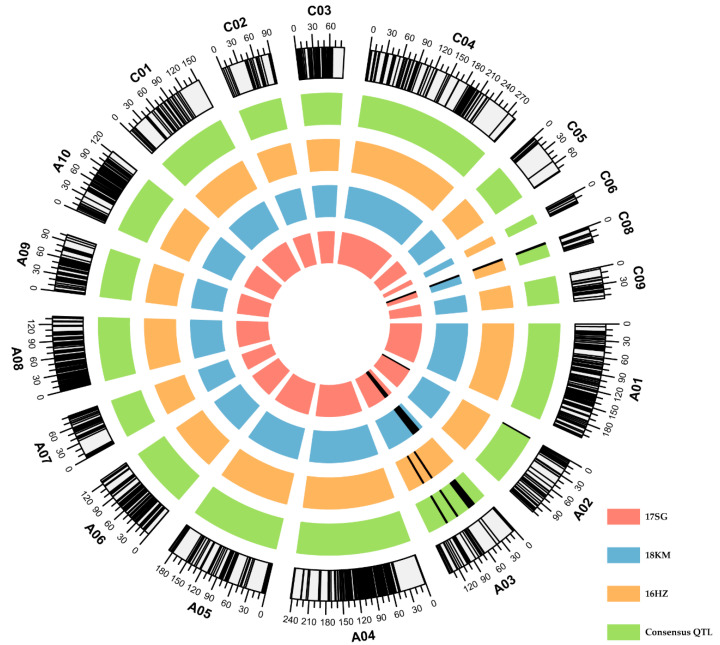
Distribution of markers and position of flowering-time quantitative trait loci (QTL) on linkage groups. Black bars in outermost circle of each block represent marker distribution on linkage groups, numbers outside each block represent linkage group genetic distances (cM). Letters plus numbers indicate linkage group number. Black bars on four circles from the inside to the outside represent QTLs identified in three environments (17 SG, 18 KM, and 16 HZ) and the consensus QTLs obtained from QTL meta-analysis. Black bar range represents QTL confidence intervals.

Brassica 60 K SNP array and SSR markers were used for genotyping the DH population to construct a high-density genetic linkage map. A total of 13,889 markers showed polymorphism among parents for further analysis. Finally, 2144 SNPs and 33 SSR markers were used to construct a genetic linkage map. The 2177 markers were separated into 18 genetic linkage groups, covering the 18 *Brassica napus* chromosomes, except chromosome C07. Among the SNPs used to construct the genetic linkage map, the physical positions of 49 SNPs were located on chromosome C07, but the linkage relationship of these SNPs was insufficient to complete the map. The average distance between adjacent markers in the genetic linkage map was 1.035 cM. There were 1368 (62.84%) SNPs located in the A subgenome, with a total length of 1455.254 cM. There were 809 (37.16%) SNPs in the C subgenome with a total length of 798.06 cM (Table S4, Figure 2). These findings showed that the A subgenome exhibited higher genetic polymorphism than the C subgenome. The average distance between markers in the A subgenome (1.064 cM) was slightly greater than in the C subgenome (0.986 cM) (Table S4), indicating that the marker density of the C subgenome was higher than that of the A subgenome.

### 2.3. Flowering-Time Quantitative Trait Loci (QTL) Mapping

QTL mapping analysis was completed based on the DH population’s flowering time data and the constructed genetic linkage map. We identified eight QTLs in three environments (logarithm of odds (LOD) threshold = 3.26). These QTLs were distributed in chromosomes A02 (one QTL), A03 (four QTLs), and C08 (three QTLs), which explained 5.32–27.87% of phenotypic variation (PVE), and additive effects from −11.87 to 9.27. Among the eight QTLs identified, five were detected in the semi-winter type region. Using QTL meta-analysis, the identified QTLs with overlapping confidence intervals from different environments were integrated to obtain five consensus QTLs; two of which (cqFT.A03-1, and cqFT.C08-1) were detected in more than one environment that explained more than 10% of the PVE in at least two environments and were considered major QTLs. cqFT.A03-1 was only detected in the semi-winter type region, and it explained 10.20% and 11.06% of the PVE. cqFT. C08-1 was detected in all environments, explaining 5.32–27.87% of the PVE (Table 2). Three of the five consensus QTLs (cqFT.A02-1, cqFT.A03-2, and cqFT.A03-3) had positive additive effects, indicating that these alleles were from ZS8, and the other two were from B409. The positions of the consensus and identified QTLs on the genetic linkage map are shown in Figure 2 and Table 2. The five consensus QTLs were compared with the published flowering-time QTLs in rapeseed. cqFT.A03-1 was mapped in the overlap genomic region with cqFT.A3-2 from Li et al. (2018) [23] and the qFT3-3 from Long et al. (2007) [24]. However, none of the other four consensus QTLs were colocalized with the QTLs reported for flowering time, indicating that they may be novel flowering-time QTLs.

### 2.4. Gene Expression and Differential Expression Analysis in RNA Sequencing (RNA-Seq) Data 

We performed RNA-seq of the parents at seven time-points in a day to enhance the efficacy of QTL mapping to predict candidate genes. After quality control of approximately 1000.14 million raw reads (~300.04 Gb bases) from 42 samples, we obtained approximately 984.44 million clean reads (~295.34 Gb bases). The clean reads were aligned to the *Brassica napus* reference genome using HISAT2. The percentage of reads that uniquely mapped to the reference genome ranged from 82.71% to 84.23% in the clean reads (Table S5). The Pearson correlation coefficient between biological replicates was high, ranging from 96.3% to 99.9% at seven time-points (Table S6, Figure S2). These results indicated that our RNA-seq data were highly reliable and could be used for subsequent analysis. The numbers of genes expressed in B409 and ZS8 ranged from 52,095 to 53,630 at seven time-points. Except for the T1 and T6 time-points, the number of genes expressed in B409 was higher than ZS8 (Figure S3A). The number of genes commonly expressed in B409 and ZS8 accounted for 91.97–93.89% of the total number of genes expressed in B409 or ZS8 (Figure S4A). There were 44,262 genes expressed in each time point of B409 and ZS8 (Figure S4B), indicating that most of the genes expressed in B409 and ZS8 were the same, and that the difference in flowering time was probably due to the difference in gene expression.

To obtain differentially expressed genes (DEGs) in B409 compared to ZS8 (B409 vs. ZS8), we screened the results from the DESeq2 R package with an false discovery rate (FDR) ≤ 0.01 and |log_2_ fold change| ≥ 2. At each of the seven time-points, the number of upregulated genes was greater than downregulated genes, indicating that for B409 compared with ZS8, the number of genes with higher expression levels was greater than with lower expression levels (Figure S3B). In B409 and ZS8, at least 11,888 genes were differentially expressed at one time-point, and 3369 genes were differentially expressed at all time-points. The number of DEGs unique to T1 was the largest, followed by T3 and T7 (Figure S5A). These three time-points were at alternate times of day and night. 

### 2.5. Functional Classification of Differentially Expressed Genes (DEGs)

**Figure 3:**
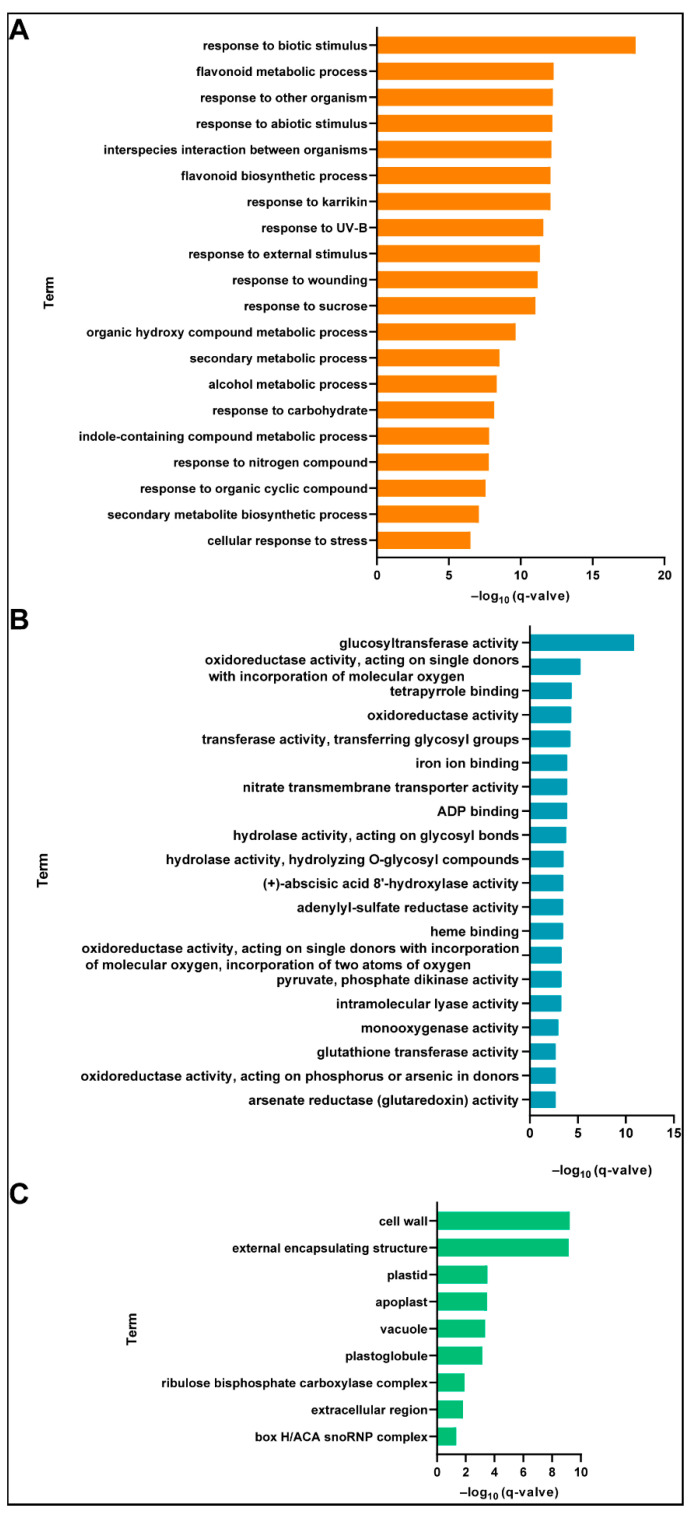
Significantly enriched Gene Ontology (GO) terms in enrichment analysis of 11,888 differentially expressed genes (DEGs). Top 20 terms with significant enrichment in biological process (**A**) and molecular function (**B**) in GO category. (**C**) Significantly enriched cellular component terms. GO enrichment analysis was performed using TBtools V1.076; *q*-value is corrected *p*-value using Benjamini–Hochberg’s method.

To explore the important biological functions of the 11,888 DEGs, we performed Gene Ontology (GO) enrichment analysis and obtained 843 GO terms, from which 441 significantly enriched terms were obtained by screening for *q*-value < 0.05. Due to the large number and redundancy of terms, we used REVIGO to remove redundant terms, obtaining 123 representative terms. Among them, there were 75 (60.97%), 39 (31.71%), and 9 (7.32%) terms for biological process, molecular function, and cellular component, respectively (Table S7). In biological process, the terms related to “metabolic” (20 terms) and “response” (23 terms) were the most abundant among the significantly enriched terms. The top five terms with the most significant enrichment were “response to biotic stimulus,” “flavonoid metabolic process,” “response to other organism,” “response to abiotic stimulus,” and “interspecies interaction between organisms” (Figure 3A). Regarding molecular function, terms related to “enzyme activity” were the most abundant, with 26 terms accounting for 66.6% of the total. The top five terms with the most significant enrichment were “glucosyltransferase activity,” “oxidoreductase activity, acting on single donors with incorporation of molecular oxygen,” “tetrapyrrole binding,” “oxidoreductase activity,” and “transferase activity, transferring glycosyl groups” (Figure 3B). In the cellular component, nine terms were significantly enriched. The top five terms with the most significant enrichment were “cell wall,” “external encapsulating structure,” “plastid,” “apoplast,” and “vacuole” (Figure 3C).

**Figure 4:**
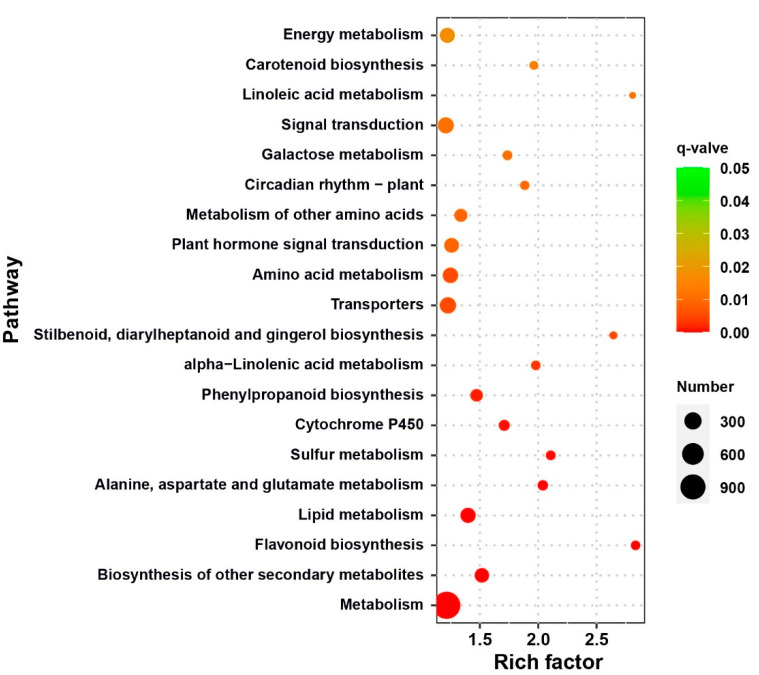
Bubble chart of top 20 Kyoto Encyclopedia of Genes and Genomes (KEGG) pathways with significant enrichment of 11,888 DEGs. Rich factor indicates degree of enrichment. Redder *q*-value color indicates a greater significant difference, and bubble size indicates number of DEGs.

We performed Kyoto Encyclopedia of Genes and Genomes (KEGG) enrichment analysis to determine the biological pathways involved in the 11,888 DEGs. Using a *q*-value < 0.05 as the threshold, we obtained 36 significantly enriched KEGG pathways, of which most (26) were in the “metabolism” class (Table S8). The 11,888 DEGs were mainly significantly enriched in “metabolism,” “biosynthesis of other secondary metabolites,” “lipid metabolism,” “cytochrome P450,” “phenylpropanoid biosynthesis transporters,” “plant hormone signal transduction,” “plant circadian rhythm,” and “signal transduction” (Figure 4). Jian et al. (2019) analyzed flowering time-related DEGs through KEGG enrichment and found that “phenylpropanoid biosynthesis transporters” and “plant hormone signal transduction” were significantly enriched in the top 20 pathways, suggesting that these two pathways may affect rapeseed flowering time [16].

### 2.6. Candidate Flowering-Time Genes Analysis

Among the DEGs between parents, we focused on those in the QTL intervals to obtain candidate genes related to flowering time. Among the 1652 genes in all QTL intervals, 289 were differentially expressed in B409 vs. ZS8. Functional annotation of the DEGs was conducted using the *A. thaliana* genome (http://www.arabidopsis.org/, last accessed date 10 January 2020). In the 289 DEGs, 282 DEGs (97.58%) had functional annotation in *A. thaliana* (Table S9), four genes of which may be flowering-time candidate genes. In cqFT.A03-1, *BnaA03g03410D* and *BnaA03g04040D* were related to flowering time and were negative regulators in the photoperiod pathway (Table 3). *BnaA03g03410D* is orthologous to *A. thaliana EMF1* (*EMBRYONIC FLOWER 1*) and the *emf1* mutant has an early flowering phenotype [25]. *BnaA03g04040D* is orthologous to *A. thaliana NF-YA1* (*NUCLEAR FACTOR Y*, *SUBUNIT A1*). Overexpression of *NF-YA1* causes late flowering under long-day conditions [26]. *BnaA03g30130D* was located in cqFT.A03-3 (Table 3). *BnaA03g30130D* is orthologous to *A. thaliana COL9* (*CONSTANS-LIKE 9*), reducing *FLOWERING LOCUS T* (*FT*) expression by inhibiting the *CONSTANS* (*CO*) expression [27]. *BnaC08g01530D* (located in cqFT.C08-1) is an ortholog of *A. thaliana AT1G05835*, but *AT1G05835′s* function has not yet been reported (Table 3); however, it encodes a PHD finger protein, and through an analysis of its protein interaction network (https://string-db.org/cgi/input.pl, using the amino acid sequence from *AT1G05835*, last accessed date 9 February 2021), we found it may be involved in *FLC* gene chromatin remodeling (Table S10, Figure S5B).

Based on the classification of the *A. thaliana* homologs of these candidate genes in FLOR-ID (http://www.phytosystems.ulg.ac.be/florid/, last accessed date 28 August 2020) and current research results, we found the three candidate genes with known function are all involved in the photoperiod pathway of flowering time regulation. A flowering-time gene could play a role in multiple flowering-time pathways. For instance, *BnaA03g03410D* is involved in the photoperiod and autonomous pathways (Table 3). *BnaA03g30130D* was detected as a DEG at one time-point only, while the other three candidate genes were detected in at least two time-points (Table S11).

### 2.7. Flowering-Time Related DEGs Analysis

**Figure 5:**
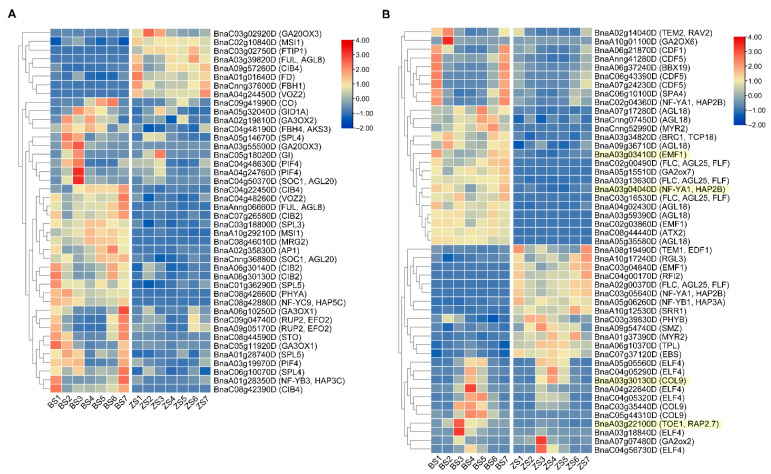
Heat maps of flowering-time genes differential expression in the photoperiod pathway. Positive and negative flowering-time regulators are indicated by (**A**,**B**), respectively. Gene names with yellow background are candidate genes in QTL intervals.

According to the functional annotation information of 11,888 DEGs in *A. thaliana*, we found that 175 DEGs were related to flowering time. We further studied which flowering-time regulation pathways were related to the difference in flowering time between B409 and ZS8. Using the classification information of the FLOR-ID website [7] and the functional study of genes. We found 175 DEGs were distributed in seven flowering-time regulatory pathways, some of which were involved in more than one pathway. Among the 175 genes, 91 were involved in the photoperiod pathway, followed by 48 and 32 in the autonomous and vernalization pathways, respectively (Figure S6A). This finding implied that the difference in flowering time between B409 and ZS8 might be due to the gene variation in these pathways, especially the photoperiod pathway. The heat map of the flowering-time gene expression in the photoperiod pathway (Figure 5) showed that the difference in gene expression was mainly reflected in the same expression pattern but different expression level (such as *BnaA06.CDF1*, *BnaC05.GI*) and difference in expression pattern (such as *BnaA02.TEM2* and *BnaC09.CO*). Based on the gene expression patterns at seven time-points in a day, the paralogs of flowering-time genes in the same material were different, such as *PIF4* and *ELF4* (Figure 5), indicating that the function of paralogs may be differentiated. In B409 compared to ZS8, the differential expression among paralogs were different, such as downregulation of *BnaA09.CIB4*, but upregulation of *BnaC04.CIB4* and *BnaC08.CIB4* (Figure 5A), suggesting that different paralogs exhibit distinct expression patterns in different materials. Gene expression in the autonomous, vernalization, and GA pathways (Figures S6B and S7) followed similar laws to the photoperiod pathway, among paralogs exhibiting different expression patterns in same or different materials, further illustrating the complexity of flowering-time traits in B409 and ZS8.

### 2.8. Quantitative Real-Time Polymerase Chain Reaction (qRT-PCR) Verification of RNA-Seq Data 

**Figure 6:**
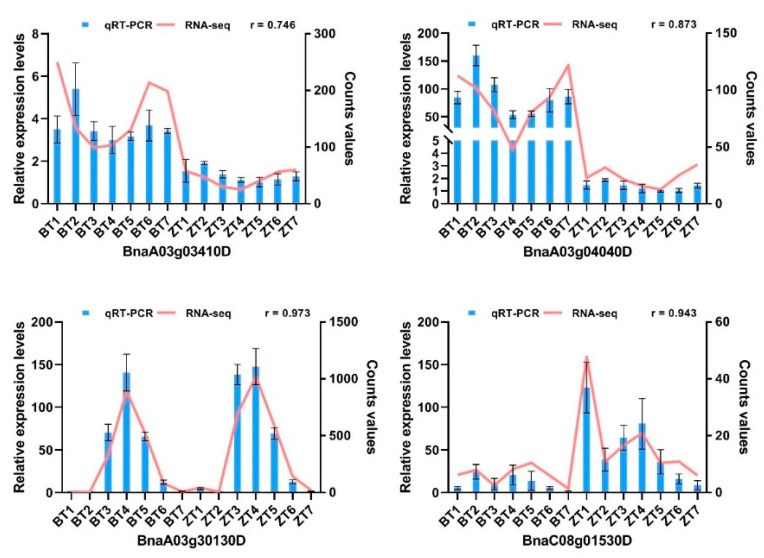
qRT-PCR validation of four candidate gene expression levels. Column charts illustrate relative gene expression levels (mean ± SE) obtained by qRT-PCR. Line charts illustrate gene expression counts values obtained by RNA-seq. r represents the Pearson correlation coefficient of gene expression obtained by qRT-PCR and RNA-seq.

We performed qRT-PCR experiments on four candidate genes and 11 randomly selected DEGs to verify the reliability of the RNA-seq results, and obtained the relative expression levels of 15 genes in B409 and ZS8. We found that the expression patterns of the genes obtained via qRT-PCR and RNA-seq at the seven time-points were highly correlated. The Pearson correlation coefficients were between 74.6% and 97.5%, with an average of 89.5% (Figure 6 and Figure S8), indicating that qRT-PCR and RNA-seq results were highly correlated, further confirming that the RNA-seq results were reliable. 

## 3. Discussion

Flowering time is a complex quantitative trait regulated by the interaction of external environmental factors (such as light and temperature) and endogenous signals [2]. Compared with the whole genome, the number of flowering time gene homologues is more, and the ratio of expression for flowering time gene homologues is also higher [18,28], complicating flowering-time regulation research in rapeseed. There are many reports on flowering time in rapeseed [23,29]; however, there are few reports on flowering-time analysis using RNA-seq assisted QTL mapping, and especially on multiple sampling in a day. Our study analyzed the DH population flowering time in different growing regions, and combined with the parental RNA-seq analysis at seven time-points in a day, four potential flowering-time candidate genes were identified. The RNA-seq data provides support for the study of the functional differentiation of paralogous genes and gene expression regulatory networks in rapeseed.

### 3.1. Flowering-Time QTLs Analysis in the DH Population

In our study, B409 flowered earlier than ZS8 in the spring-type region and ZS8 flowered earlier than B409 in the semi-winter region. The semi-winter type region had shorter day length and longer vernalization time than the spring type region, and B409 was more sensitive to photoperiod than the other materials (Table S1). Therefore, we speculated that the difference in B409 and ZS8 flowering time might have been due to the difference in photoperiod response. A total of 2177 markers were used to construct a genetic linkage map, with an average distance of 1.035 cM between markers (Table S4). This marker density was slightly lower than in other studies using the Brassica 60 K SNP array [9,16]. There was large marker spacing between some positions, resulting in a large QTL interval (as cqFT.A03-1). Whole-genome resequencing could be used to develop additional molecular markers to construct local high-density genetic linkage maps for candidate interval.

Two consensus QTLs were detected as major QTLs in at least two environments. cqFT.A03-1 was only detected in the semi-winter type region and they may be related to the photoperiod and vernalization pathways. cqFT.A03-1 was a region-specific QTL detected in all semi-winter type region. The study of region-specific QTL is beneficial for selecting rapeseed varieties suitable for planting in specific region. cqFT.C08-1 existed stably in all environments but it has not been reported in the current study; it may be a novel flowering-time QTL. The detection of stable QTL in different regions is conducive to breeding varieties with broad adaptability. The remaining two QTLs were detected only in 16 HZ and they may be environment-specific. These two major QTLs contained large PVEs and additive effects, conducive to fine mapping and gene cloning, and provide a genetic basis for improving rapeseed flowering time.

### 3.2. Candidate Flowering-Time Genes Analysis in QTL Intervals 

In the cqFT.A03-1 interval, both flowering-time-related candidates were involved in photoperiod pathway (Table 3). cqFT.A03-1 was detected specifically in the semi-winter type region, implying that it may be a photoperiod-related QTL. BnaA03g03410D (EMF1) protein can form a polycomb group (PcG) complex (EMF1C) that inhibits *FT* expression. Simultaneously, EMF1c and CO have antagonistic effects on *FT* chromatin binding [30]. *BnaA03g04040D* (*NF-YA1*) belongs to the *NF-YA* transcription factor family. The overexpression of *NF-YA1* causes late flowering, and the NF-YA1 protein might replace the CO protein to form a trimeric complex with NF-YB and NF-YC that inhibits *FT* expression [26,31]. No DEGs in cqFT.C08-1 were related to flowering time; however, the *BnaC08g01530D* (PHD finger protein) function was not clear. It may have been involved in *FLC* chromatin modification (Table S10, Figure S5B). It should be overexpressed and gene-edited to determine whether its function is related to flowering time. *BnaA03g30130D* (*COL9*) belongs to the *CO* gene family; it reduces *FT* expression by inhibiting *CO* expression [27]. Consistent with previous studies [32,33], we found that the combination of QTL mapping and RNA-seq could effectively predict candidate genes.

The three candidate genes with known functions were negative regulators in the photoperiod pathway. They mainly participate in photoperiod regulation by competitively binding with CO protein to the *FT* promoter or by inhibiting *CO* expression. In the RNA-seq analysis, we found that *BnaA09.CO* was differentially expressed in the parents (Figure 5). The number of genes in the photoperiod pathway was the largest among the differentially expressed flowering-time genes, indicating that the difference in flowering time between the parents was closely related to the photoperiod pathway. 

### 3.3. Advantages of RNA-Seq Analysis at Multiple Time-Points in a Day

The circadian clock is a ubiquitous biological system in plants that regulates several important plant development processes, including flowering [34]. Owing to the circadian clock and environmental signals such as day length, the expression patterns of some genes show rhythmic daily oscillations, including many flowering-time genes in the photoperiod pathway [34,35], which complicates the selection of a particular RNA-seq sampling time. Day length is perceived by leaves and it modulates *FT* expression to regulate flowering [36]. Therefore, in this study, we sequenced parent leaves in the early flowering response at seven time-points in a day to obtain the DEGs, and combined them with the QTL results to identify flowering-time candidate genes. However, in several previous studies, RNA-seq was only performed on samples taken at one time in a day. Jian et al. (2019) realized that sampling time significantly affects gene expression changes in the photoperiod pathway [16]. None of our four candidate genes were differentially expressed at seven time-points of a day (Table S11); therefore, if only one time-point is sampled, it is likely that important candidate genes could be missed. Therefore, our multiple time-point sampling method for RNA-seq was instrumental in discovering potential candidate genes in the QTL intervals that might not otherwise have been identified.

In our RNA-seq, the correlation coefficient between biological repeats at the seven time-points was as high as 96.6% to 99.9% (Table S6), indicating that it could more veritably reflect the gene expression model in a day. Similar diurnal transcriptome studies in animals [19,37] and plants [22,38] have been completed, and it has been reported that changes in the diurnal expression of genes may be related to adaptability [22,37]. Therefore, our RNA-seq data can show the dynamic expression network of genes in rapeseed during a day. This is helpful for research on the functional differentiation of paralogous genes in rapeseed, and provides data support for the adaptability research of polyploid crops.

## 4. Materials and Methods

### 4.1. Plant Materials and Growth Conditions

A 148-line DH population was constructed from a cross between Bing409 (B409) and Zhongshuang8hao (ZS8). The DH lines and their parents were planted in the experiment field using a randomized complete block design with two replications at three locations in China. A combination of year and location was used to name the environments. For example, 17 SG indicates that the data was from Shaoguan in the 2016–2017 growing season. The environment information for 17 SG, 18 KM, and 16 HZ is shown in Table S12. SG, and KM were semi-winter type rapeseed growing region; the seeds were sown in October and harvested in May. HZ was spring type rapeseed growing region; the seeds were sown in April and harvested in August. Each plot in each environment consisted of one row, with approximately 12 plants with a 30 cm in-row spacing. The monthly average day lengths of the three environments were shown in Figure S1A, and the monthly average minimum and maximum temperatures of the three environments are shown in Figure S1B,C, respectively. 

### 4.2. Phenotype Data Measurement and Analysis

Flowering-time data were the number of days from the sowing date to 25% of the rapeseed plants exhibiting at least one open flower in a plot [4]. The average flowering time of two replicates of the DH population in each environment was used as the phenotypic value for subsequent analysis. GraphPad Prism 8 (GraphPad Software, Inc., San Diego, CA, USA) was used to analyze the significance of the difference in the flowering time of the parents (using the unpaired *t*-test) and the correlation between three environments (using the paired *t*-test). The average, standard deviation, skewness, and kurtosis values of the three environmental flowering times were obtained from QTL IciMapping V4.2 [39]. IBM SPSS Statistics 26 software was used to perform a two-way analysis of variance on flowering time [40].

### 4.3. Genotyping and Markers Filtering

The 148 lines and parents of genomic deoxyribonucleic acid (DNA) were extracted from young leaf tissue of three plants in each material, using the cetyltrimethylammonium bromide method [41] to obtain SNP genotyping data from the DH population and parents. The quality of genomic DNA was detected on 1% agarose gel, and the concentration was determined using a NanoDrop spectrophotometer (Thermo Fisher Scientific, Wilmington, DE, USA). SNP genotyping of the DH population and parents was performed using the Brassica 60 K Illumina Infinium™ SNP array with 52,157 SNPs, according to the manufacturer’s instructions [42]. The scan data were imported into Genome Studio software (Illumina Inc., San Diego, CA, USA) to obtain the SNP genotype data. We also used SSR markers for the DH population genotyping. The Cai et al. (2015) method was used to filter the SSR and SNP genotyping results [43]. The lines with heterozygous site frequencies > 10% were eliminated to avoid mixing plants from the non-DH population. To obtain effective markers to construct a genetic linkage map, the following four marker types were eliminated: no polymorphism between parents, non-parental genotypes, excessive missing SNPs (>10%), and distorted segregation (>5%). 

### 4.4. Genetic Linkage Map Construction and QTL Mapping

A total of 2177 markers containing 2144 SNPs and 33 SSR markers for the DH population were used to construct a genetic linkage map. The linkage groups were constructed using JoinMap 4.0 software [44]. The genetic distance between markers was calculated using the Kosambi mapping function. QTL IciMapping V4.2 software was used to detect the QTLs for flowering time via inclusive composite interval mapping (ICIM) [39]. The ICIM-ADD mapping method software with BIP functionality was used to detect a single environment QTLs. QTLs from the three environments were meta-analyzed using BioMercator V4.2 software to obtain consensus QTLs [45]. The LOD threshold for detecting a significant QTL was established by permutation analysis with 1000 permutations, using the default settings. The QTL that explained more than 10% phenotypic variation in at least two environments were considered major QTL. Circos plots displaying a genetic linkage map and QTL locations were completed using TBtools V1.076 software [46].

### 4.5. Leaf Sampling and RNA Isolation

B409 and ZS8 seeds were sown separately in 9 × 9 cm pots containing a mixture of peat, nutrition substance, and vermiculite in a 5:4:1 volume ratio and placed in an AR75L growth chamber (Percival Scientific Inc., Perry, IA, USA) with an 8 h light/16 h dark diurnal cycle. The growth chamber was maintained at a constant temperature of 22 °C and 65% relative humidity. The fourth true leaves of B409 and ZS8 were sampled every 4 h for 1 day at 30 days after sowing (Figure S1D). The first sampling was conducted as soon as the light was turned on. Three biological replicates were obtained per time point, and each biological replicate was composed of four independent plants. The samples were immediately snap-frozen in liquid nitrogen and stored at −80 °C. Total RNA was isolated from 42 samples using an RNAprep Pure Plant Plus Kit (Polysaccharides & Polyphenolics-rich) (Tiangen Biotech Co. Ltd., Beijing, China) according to the manufacturer’s instructions. The RNA quality was detected by 1% agarose gel electrophoresis, and its concentration and purity were determined using a NanoDrop spectrophotometer (Thermo Fisher Scientific, Wilmington, DE, USA). 

### 4.6. RNA-Seq and Data Analysis

cDNA library construction and sequencing were performed by Novogene Bioinformatics Technology Co., Ltd. (Tianjin, China). The RNA integrity was detected using an Agilent 2100 Bioanalyzer (Agilent Technologies, Palo Alto, CA, USA). Total RNA (1 µg) from each sample was used to construct a cDNA library, using a NEBNext^®^ UltraTM RNA Library Prep Kit (New England Biolabs, Inc., Ipswich, MA, USA) according to the manufacturer’s instructions. Subsequently, 42 libraries were sequenced on an Illumina NovaSeq 6000 sequencer (Illumina, Inc., San Diego, CA, USA) to obtain 150 bp paired-end reads. The raw reads were filtered to obtain clean reads by removing reads with an N content > 10% of the base number of reads and low-quality (Q <= 5) base number reads > 50% of the base number of the reads, using fastp software [47]. The clean reads were aligned to the “Darmor-*bzh*” reference genomes [1] using HISAT2 [48], and the uniquely mapped reads were obtained. Subsequently, featureCounts software was used to count the unique reads [49]. Genes with >10 counts from the sum of three biological replicates were considered expressed genes [15]. The pheatmap R package was used to draw a correlation heat map between the samples (https://github.com/raivokolde/pheatmap, last accessed date 7 December 2020). The DESeq2 R package was used to generate normalized read counts and DEG analysis [50]. The DEGs were defined by a FDR ≤ 0.01, and the absolute value of log_2_ fold change ≥ 2 between samples. A Venn diagram was drawn using TBtools V1.076 software [46], and an UpSet Venn diagram was drawn using OmicShare tools (http://www.omicshare.com/tools, last accessed date 6 January 2021). The GO annotation of the Darmor-*bzh* genes was obtained using Blast2GO [51] and InterProScan 5 [52]. TBtools V1.076 software was used to perform GO and KEGG enrichment analysis of the DEGs [46]. The terms with a corrected *p*-value (*q*-value) < 0.05 (using the Benjamini–Hochberg method) were considered significantly enriched in the DEGs. The representative GO terms were obtained using REVIGO (http://revigo.irb.hr/, last accessed date 8 December 2020) to summarize the redundant GO terms [53]. TBtools V1.076 software was used to draw a gene-expression heat map [46].

### 4.7. qRT-PCR Validation of RNA-seq Data

To determine the RNA-seq data reliability, we selected four candidate genes and randomly selected 11 DEGs for qRT-PCR analysis. The RNA samples from RNA-seq were used to synthesize first-strand cDNA by RevertAid First Strand cDNA Synthesis Kit (Thermo Fisher Scientific, Waltham, MA, USA) according to the manufacturer’s instructions. The sequence alignment of gene copies was used to design the specific qRT-PCR primers. The *ACTIN2* gene was used as the internal control. qRT-PCR was performed using a ChamQ Universal SYBR^®^ qPCR Master Mix (Vazyme Biotech Co. Ltd., Nanjing, China) in a Bio-Rad CFX384^TM^ Real-Time System (Bio-Rad, Hercules, CA, USA). The qRT-PCR primers are illustrated in Table S13. The qRT-PCR for each gene was completed using three biological replicates, and each biological replicate contained three technical replicates. The relative gene expression levels were calculated by the 2^−ΔΔCT^ method [54].

## Figures and Tables

**Table 1:** Phenotypic variation of flowering time in the double haploid (DH) population and their parents.

Environment	Parents		DH Lines			
	B409 ^a^	ZS8 ^a^	DH Lines Range	Mean ± SD	Skewness	Kurtosis
17 SG	131 ± 2.94	106.6 ± 4.13 ***	60–159	114.47 ± 22.95	−0.59	−0.52
18 KM	133.43 ± 0.49	120.43 ± 0.73 ****	100.5–139	126.03 ± 7.21	−0.59	1.49
16 HZ	68.44 ± 1.57	71 ± 2.24 ***	64–87.3	74.73 ± 5.07	0.36	−0.61

The significance level by *t* test: *** *p* < 0.001, **** *p* < 0.0001. ^a^ Mean ± SD, SD means standard deviation.

**Table 2:** Summary of the consensus QTLs and identified QTLs in three environments.

Consensus QTLs				Identified QTLs						
QTLs	Chr. ^a^	CI (cM) ^b^	PI (kb) ^c^	QTLs	Peak (cM)	CI (cM) ^b^	LOD ^d^	PVE (%) ^e^	Add ^f^	Env. ^g^
cqFT.A02-1	A02	0–0.5	5672–5851	qFT.A02-1	0	0–0.5	8.30	16.21	9.27	17 SG
cqFT.A03-1	A03	26.33–40.39	574–4966	qFT.A03-1	34	22.5–39.5	5.46	10.20	−6.86	17 SG
				qFT.A03-2	32	14.5–39.5	4.51	11.06	−2.43	18 KM
cqFT.A03-2	A03	69.5–71.5	9834–10,261	qFT.A03-3	71	69.5–71.5	11.82	17.56	2.82	16 HZ
cqFT.A03-3	A03	95.5–97.5	12,981–15,100	qFT.A03-4	97	95.5–97.5	5.16	6.99	1.80	16 HZ
cqFT.C08-1	C08	0–2.5	802–1388	qFT.C08-1	1	0–2.5	13.11	27.87	−11.87	17 SG
				qFT.C08-2	2	0–2.5	9.20	24.43	−3.71	18 KM
				qFT.C08-3	0	0–0.5	4.13	5.32	−1.64	16 HZ

^a^ Chromosome. ^b^ Confidence interval. ^c^ Physical interval. ^d^ Likelihood of odd. ^e^ Phenotypic variation explained by QTL. ^f^ Additive effect of QTL. ^g^ Environment.

**Table 3:** Information on flowering time genes in QTL intervals.

QTL Name	*B. napus* ID	TAIR ID	Gene	Regulator	Pathway
cqFT.A03-1	*BnaA03g03410D* ^a^	*AT5G11530*	*EMF1*	Negative	Autonomous pathway, photoperiod pathway
	*BnaA03g04040D*	*AT5G12840*	*NF-YA*1, *HAP2B*	Negative	Photoperiod pathway
cqFT.A03-3	*BnaA03g30130D*	*AT3G07650*	*COL9*	Negative	Photoperiod pathway
cqFT.C08-1	*BnaC08g01530D*	*AT1G05835*	PHD finger proteins	Unknown	Unknown

^a^ Genes underlined indicate that they were DEGs at least at one time point in B and Z.

## Data Availability

The raw sequence data of RNA-seq in this paper have been deposited in the Genome Sequence Archive in National Genomics Data Center, under accession number CRA004526 that are publicly accessible at https://ngdc.cncb.ac.cn/gsa accessed date 9 February 2021.

## Supplementary Materials

The following are available online at https://www.mdpi.com/article/10.3390/ijms22147559/s1.

## Abbreviations

QTL	Quantitative trait loci
DH	Double haploid
ZS8	Zhongshuang8hao
B409	Bing409
DNA	Deoxyribonucleic acid
SNP	Single nucleotide polymorphism
SSR	Simple sequence repeat
ICIM	Inclusive composite interval mapping
LOD	Logarithm of odds
FDR	False discovery rate
PVE	Phenotypic variation
RNA	Ribonucleic acid
RNA-seq	Ribonucleic acid sequencing
DEGs	Differentially expressed genes
*A. thaliana*	*Arabidopsis thaliana*
GA	Gibberellin
GO	Gene Ontology
KEGG	Kyoto Encyclopedia of Genes and Genomes
qRT-PCR	Quantitative real-time polymerase chain reaction
