# Supplementary material for: QTL Mapping and Diurnal Transcriptome Analysis Identify Candidate Genes Regulating Brassica napus Flowering Time
Source: Int J Mol Sci. 2021 Jul 15;22(14):7559. doi: 10.3390/ijms22147559 (PMC8305928; doi:10.3390/ijms22147559)
Supplement: Supplementary file 1 [file ijms-22-07559-s001.zip › Supplementary Figures.pdf]

Supplementary Figures

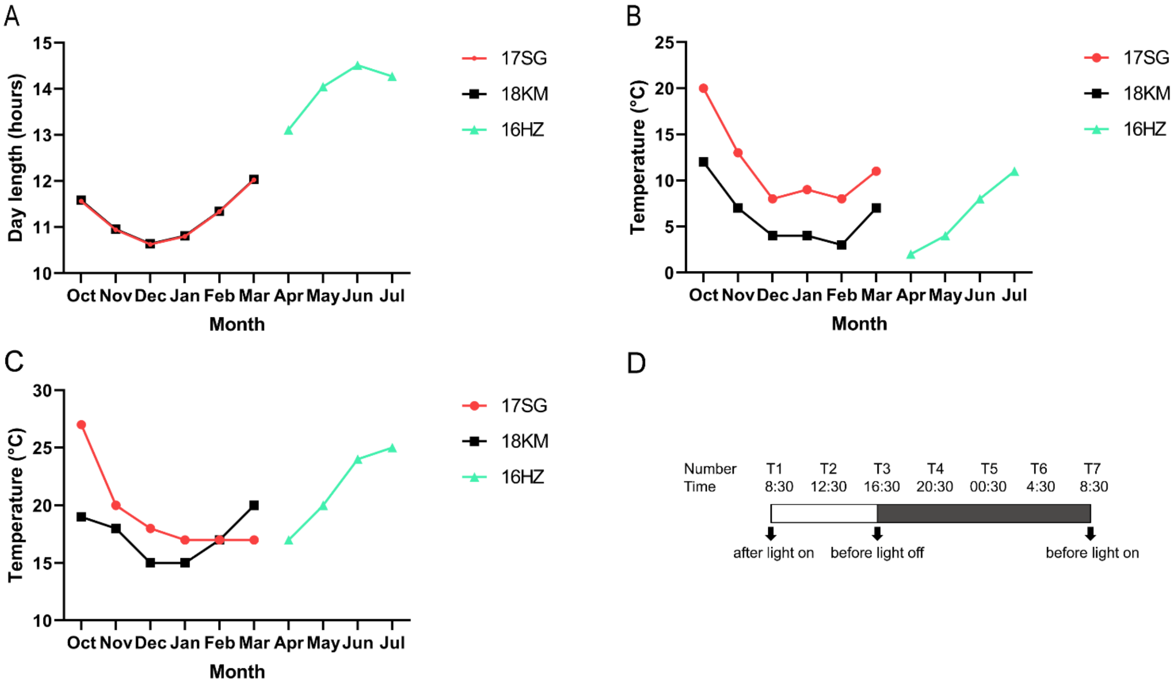

Figure S1. Average day length, temperature, and RNA-seq sampling diagram**.** Monthly average day length (**A**) and monthly average minimum (**B**) and maximum (**C**) temperature in three environments, and RNA-seq sampling diagram (**D**). Day length data was obtained from https://richurimo.51240.com/, and temperature data from http://www.tianqi.com/. T1-T7 represents seven sampling time-points during a day.

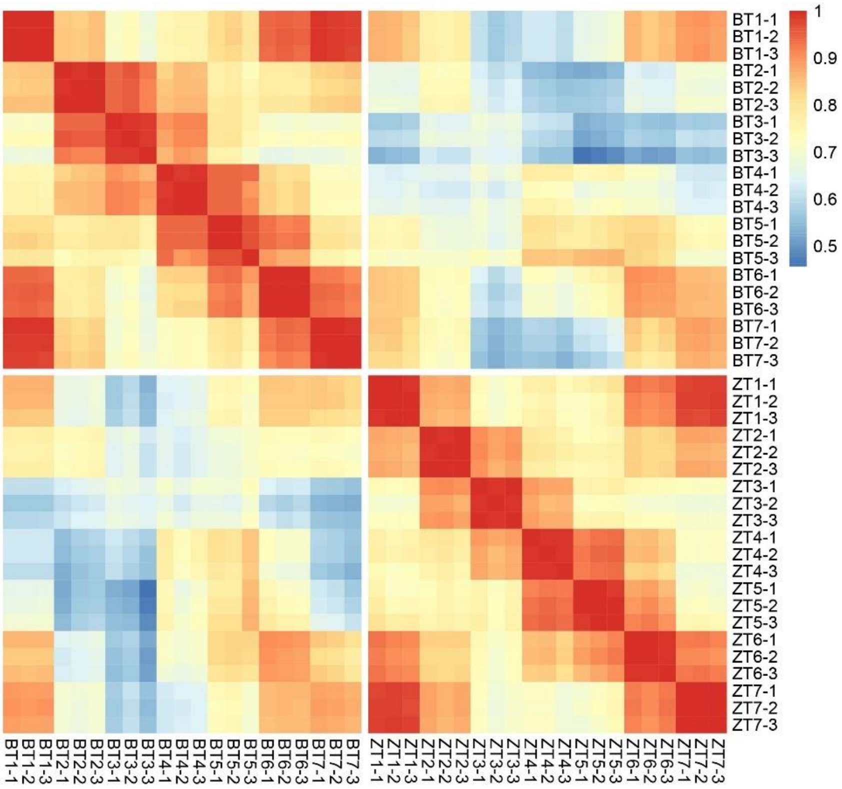

Figure S2. Heat map for correlation between RNA sequencing samples. Redder color indicates higher correlation between samples. B represents B409; Z represents ZS8; T1-T7 represent sampling time-points, -1, -2, -3 represent three biological replicates at each time-point.

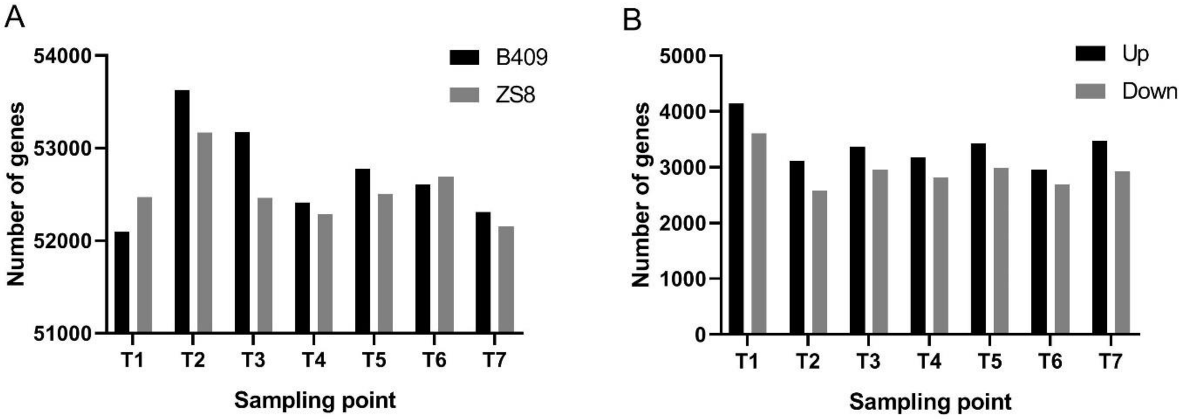

Figure S3. Column chart of gene expression. Number of genes expressed (**A**) and differentially expressed (**B**) at each time-point in RNA-seq data.

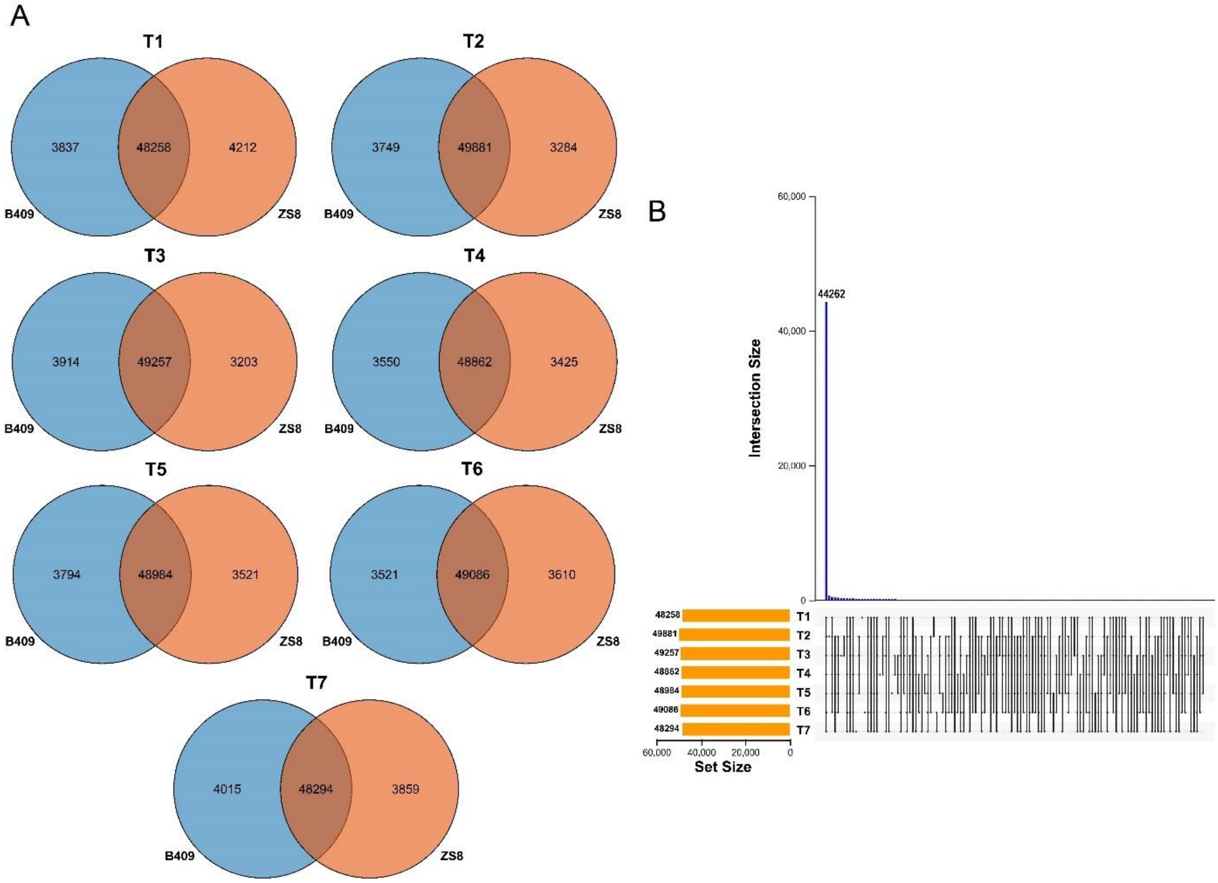

Figure S4. Venn diagram of expressed genes in B409 and ZS8. (**A**)Venn diagram of genes expressed in B409 and ZS8 at each time-point, and (**B**) UpSet Venn diagram of commonly expressed genes in B409 and ZS8 at each time-point.

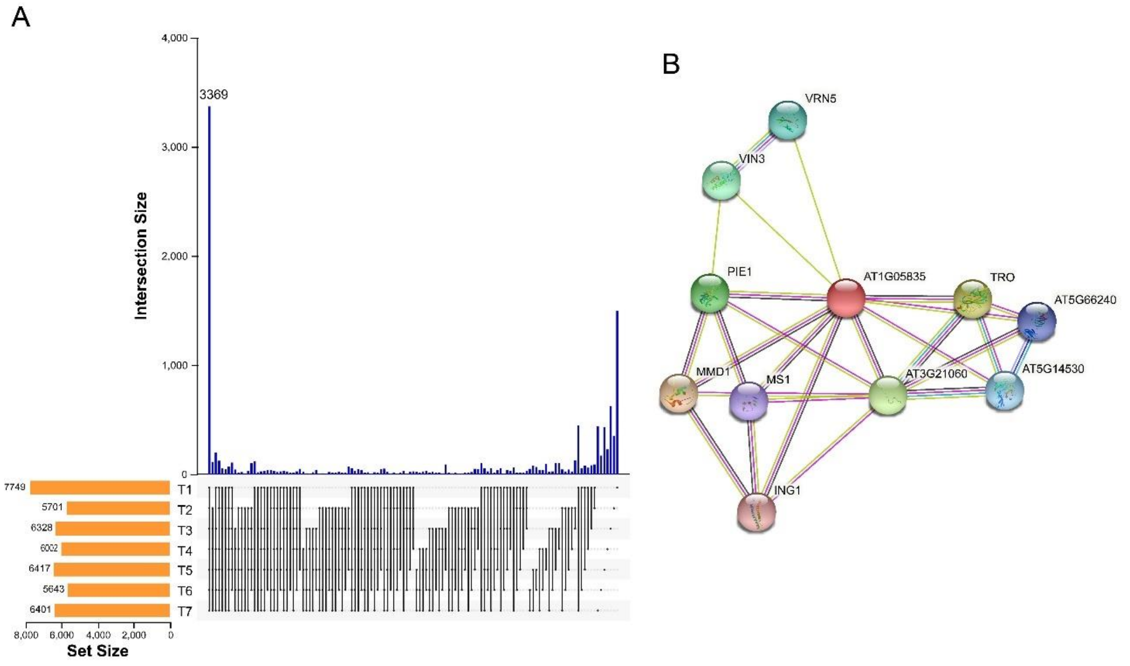

Figure S5. UpSet Venn of DEGs and protein interaction network diagrams. (**A**)UpSet Venn diagram of DEGs for B409 compared to ZS8, and (**B**) AT1G05835 protein interaction network diagram using STRING website (https://stringdb.org/cgi/input.pl, last accessed date 9 February 2021).

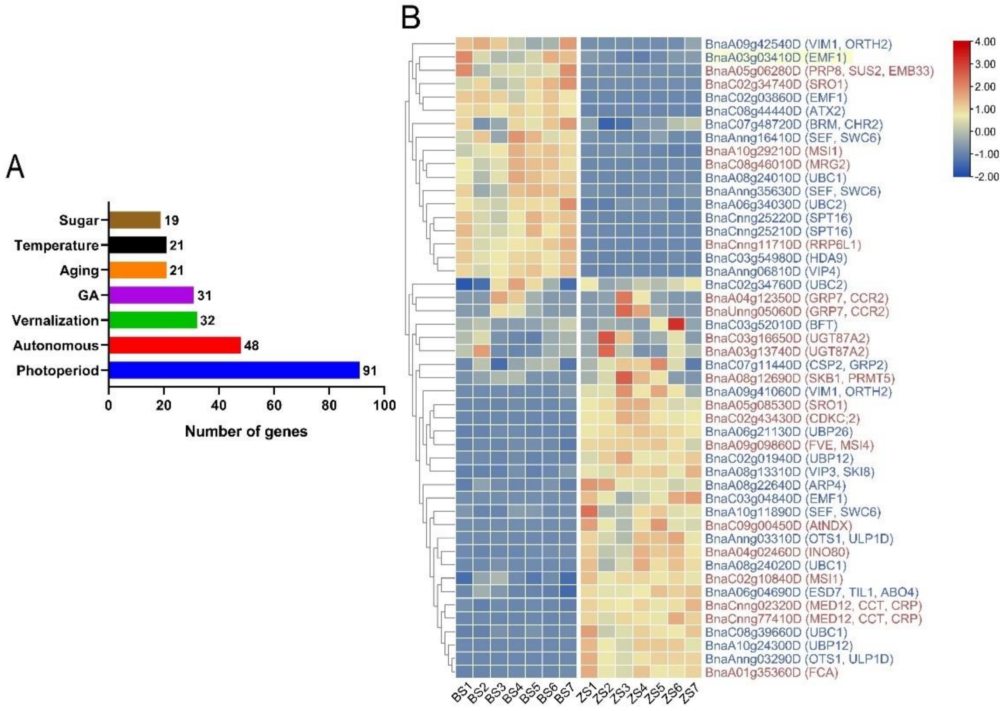

Figure S6. Histogram and heat map of differentially expressed flowering-time genes. (**A**) Histogram of number of differentially expressed flowering-time genes in seven pathways. (**B**) Heat map of differential expression of autonomous pathway flowering-time genes. Red and blue gene names indicate genes that are positive and negative flowering-time regulators, respectively. Gene name with yellow background are candidate genes in QTL intervals.

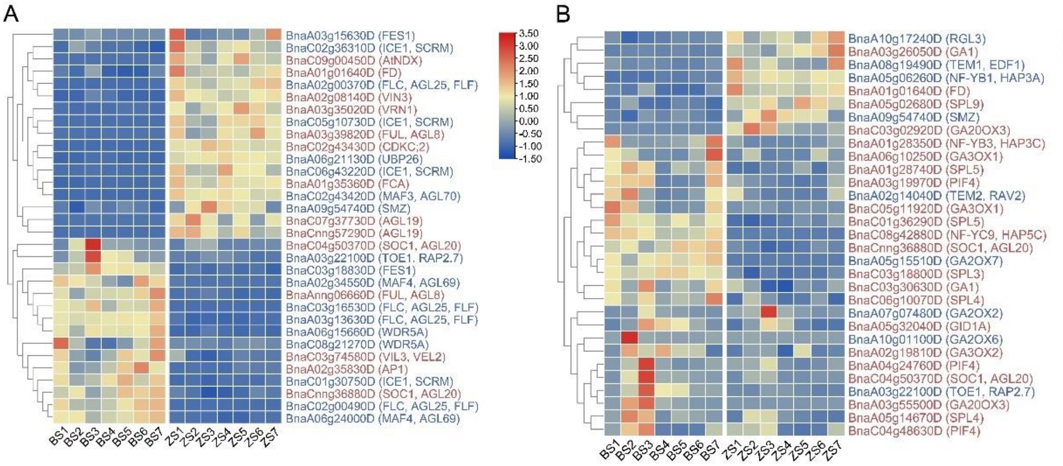

Figure S7. Heat map of differential expression of flowering-time genes in vernalization and GA pathways. Heat map of differential expression of flowering-time genes in vernalization (**A**) and GA pathways (**B**). Red and blue gene names indicate positive and negative flowering-time gene regulators, respectively. Gene names with yellow background indicate candidate genes in QTL interval.

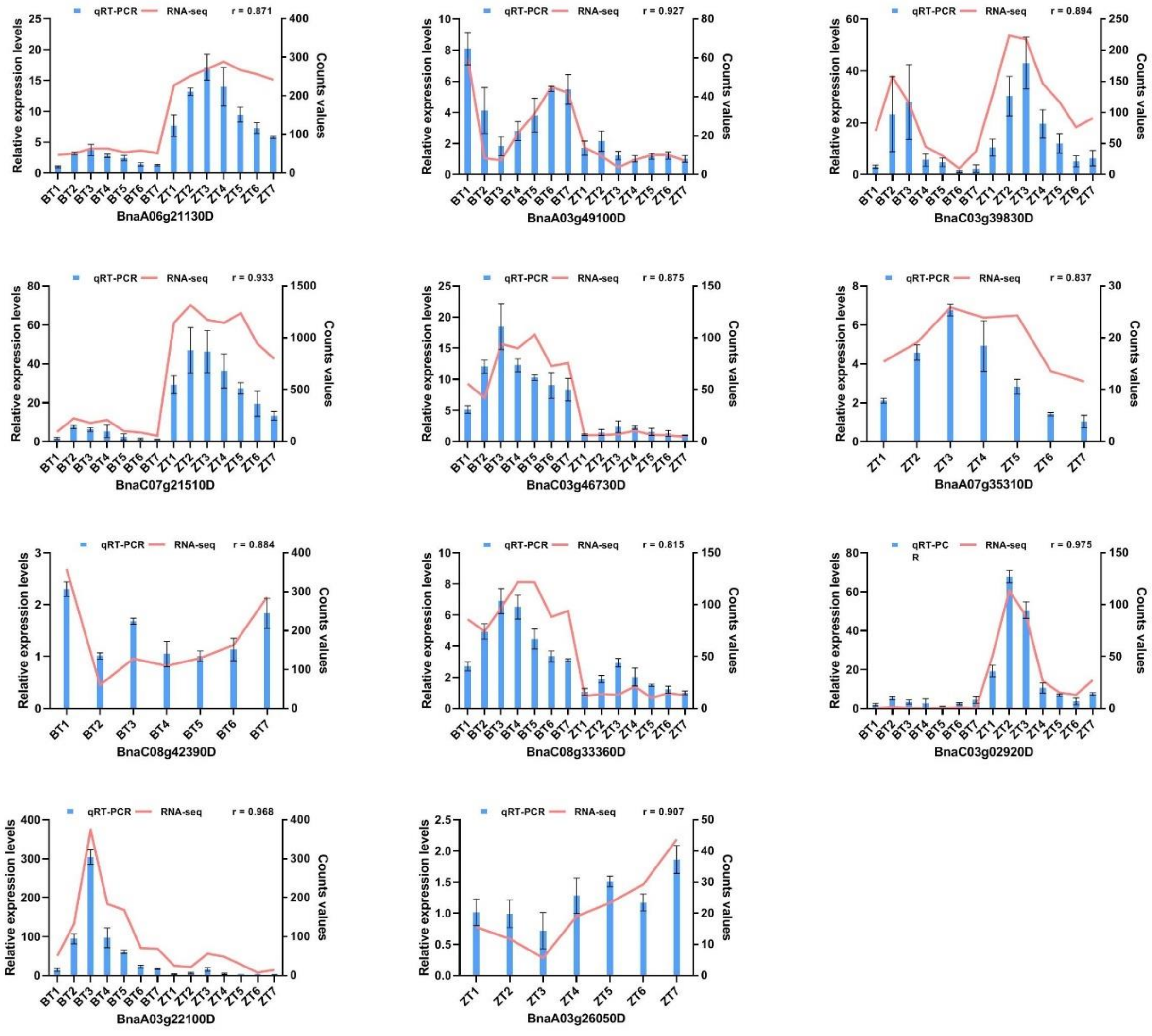

Figure S8. qRT-PCR validation of eleven randomly selected DEGs. Column charts illustrate relative gene expression levels (mean ± SE) determined via qRT-PCR. Line charts illustrate gene expression counts values obtained via RNA-seq. r represents the Pearson correlation coefficient of gene expression determined via qRT-PCR and RNA-seq.
